# Assessment of mutations on RBD in the Spike protein of SARS-CoV-2 Alpha, Delta and Omicron variants

**DOI:** 10.1038/s41598-022-12479-9

**Published:** 2022-05-20

**Authors:** Clauber Henrique Souza da Costa, Camila Auad Beltrão de Freitas, Cláudio Nahum Alves, Jerônimo Lameira

**Affiliations:** Laboratório de Planejamento e Desenvolvimento de Fármacos, Universidade Federal do Pará, Rua Augusto Correa S/N, Belém, PA Brazil

**Keywords:** Biochemistry, Proteins, Biophysics, Computational biophysics, Protein analysis, Computational chemistry, Molecular dynamics

## Abstract

The severe acute respiratory syndrome (SARS) coronavirus 2 (CoV-2) variant Omicron spread more rapid than the other variants of SARS-CoV-2 virus. Mutations on the Spike (S) protein receptor-binding domain (RBD) are critical for the antibody resistance and infectivity of the SARS-CoV-2 variants. In this study, we have used accelerated molecular dynamics (aMD) simulations and free energy calculations to present a systematic analysis of the affinity and conformational dynamics along with the interactions that drive the binding between Spike protein RBD and human angiotensin-converting enzyme 2 (ACE2) receptor. We evaluate the impacts of the key mutation that occur in the RBDs Omicron and other variants in the binding with the human ACE2 receptor. The results show that S protein Omicron has stronger binding to the ACE2 than other variants. The evaluation of the decomposition energy per residue shows the mutations N440K, T478K, Q493R and Q498R observed in Spike protein of SARS-CoV-2 provided a stabilization effect for the interaction between the SARS-CoV-2 RBD and ACE2. Overall, the results demonstrate that faster spreading of SARS-CoV-2 Omicron may be correlated with binding affinity of S protein RBD to ACE2 and mutations of uncharged residues to positively charged residues such as Lys and Arg in key positions in the RBD.

## Introduction

First reported in the city of Wuhan, China^1,2^, *Coronavirus disease* (COVID-19) named by World Health Organization (WHO) was declared a global pandemic on March 2020^3^. COVID-19 is caused by the severe acute respiratory syndrome coronavirus 2 (SARS-CoV-2)^1,2,4,5^. The spread of SARS-CoV-2 have cost millions of lives and caused many implications for health, society and the economy^6,7^. In January 2022, the WHO reported over 304 million confirmed cases of COVID-19 and over 5.4 million fatalities have been reported since the beginning of the outbreak^8^. Vaccines are effective for reducing the number deaths by COVID-19^9–11^. On the other hand, variants may cause impact on the virus recognition by antibody-mediated vaccines^12–14^.

Different mutations have been reported in the gene encoding the S protein of SARS-CoV-2^15,16^, and recently, the world have faced rapid increase in COVID-19 mediated by new variants^17^. The last variant detected was named Omicron (B.1.1.529)^18^, identified in numerous countries in November 2021, first reported in South African with a large number of mutations, including K417N, S477N, T478K, E484A, and N501Y, which are also found in other variants^19–21^ and evidences suggests there may be an increased risk of reinfection involving this variant^22,23^ due to improve viral escape or binding affinity to angiotensin-converting enzyme 2 (ACE2)^24–26^.

A recent study reported that 45 point mutations was identified and found that the Omicron Spike protein sequence was subjected to stronger positive selection than that of any reported SARS-CoV-2 variants^27^. Additionally, These mutations and deletions in the S-protein sequence can alter the structure, affecting its stability and function, further exacerbating SARS-CoV-2 infectivity^16,28^. However, N501Y mutation is a key contact residue in the receptor-binding domain (RBD), enhancing virus binding affinity to ACE2^29–31^ making the virus more contagious and the deletions H69/V70 is required for increase optimal infectivity of Alpha variant, that drives by higher levels of Spike incorporation into virions^32^.

Coronaviruses use Spike (S) glycoprotein, with S1 subunit and S2 subunit in each Spike monomer, anchored in the virion envelope to bind to their cellular receptors^33,34^ and mediates the recognition of the host-cell receptors and facilitates the cell attachment (S1 subunit) and the cell membrane fusion (S2 subunit) during the viral infection^35^. The RBD located in the S1 region (318–510 sequence region) performs strongly binds to the peptidase domain of ACE2^36,37^, leading to a critical virus-receptor interaction and reflects viral host range, tropism and infectivity^38^. The RBD of S1 undergoes conformational changes that transiently conceal or reveal the determinants of receptor binding^24,39^.

The Spike (S) protein of SARS-CoV-2 consists in an extracellular N-terminus, a transmembrane (TM) domain and a intracellular C-terminal segment^40^. S protein has a total length of 1273 amino acids^35^ and molecular weight of 180–200 kDa^41^. It has a signal peptide (1–13) at the N-terminus, followed by S1 subunit (14–685) and the S2 subunit (686–1273)^35^. The structure of the RBD allows for ways to alter its genetic material, developing variants by the changes in Spike protein amino acids and as viruses replicate^16^, copying errors of itself, resulting in mutations that arise in their genomes generating several strains of SARS-CoV-2^17,42^ that differ in transmission, infectivity and severity of the disease^42^.

ACE2 primary physiological role is in the maturation of angiotensin (Ang)^43^, a peptide hormone that controls vasoconstriction and blood pressure, is a type I membrane protein expressed in lungs, heart, kidneys, and intestine^25,44^, thereat, decreased expression of ACE2 is associated with cardiovascular diseases^45^. The structural features of RBD increase its binding affinity to the ACE2 receptor and it is a significant step for SARS-CoV-2 to enter into target cells^33,46^. Computer modelling studies of the interaction between the SARS-CoV-2 RBD and ACE2 were able to identify the residues involved in this interaction and elucidate how the structural change benefits receptor recognition and virus entry into the host cell, that occurs by proteolytic processing of the Spike protein to promote cell-virus fusion^47^. Therefore, atomic details may clarify the importance and significance of investigating the changes in residues that facilitate efficient cross-species infection and human-to-human transmission^34^. Whereas the essential evolution and consequent mutation of SARS-CoV-2 takes place remotely from the RBD in the Spike protein, such evolution may facilitate the conformational change in specific residues, punctually interfering with the infection process that occurs after the virus binds to ACE2^48^.

Recently, Warshel and co-workers studied the mechanism of the binding affinity changes for mutations at different Spike protein domains of SARS-CoV-2, Alpha, Beta and Delta variants using coarse-grained potential surface to calculate the binding free energy of SARS-CoV-2 to ACE2^49^, concluding that the evolution of the virus takes place from the binding domain in the trimeric body of the Spike protein, which may facilitate the conformational change and the infection process. Chen and co-workers used machine learning model to analyze how the RBD mutations on the Omicron variant may affect the viral infectivity and efficacy of existing vaccines and antibody drugs^50^. They results indicated that the Omicron variant may be ten times more contagious than the Wild Type (WT) virus or about twice as infectious as the Delta variant, also based on the Spike protein binding domain^50^. More recently, Kumar and co-workers^51^ molecular dynamics (MD) simulations to investigate the interaction between the RBD of both the WT and Omicron variant with the ACE2 receptor and found that the Omicron Spike protein has an increased affinity for the ACE2 receptor, due to the presence of mutant residues^51^. Similarly, Socher and coworkers have used MD simulations of the RBD and ACE2 to analyze and compare the interaction pattern between the WT, Delta and Omicron variants, where they have identified residue 493 in Delta (glutamine) and Omicron (arginine) with altered binding properties towards ACE2^52^. MD simulation have also been used to explore the effect of different possible mutations of the Spike key residues, which are the mutations found in the most relevant observed variants^53^. In this study, we have used all-atom accelerated molecular dynamics (aMD) simulations^54,55^ to explore the impacts of the substitutions that occur in the Spike RBD of Alpha, Delta and Omicron variants in the binding with the human ACE2 receptor. In order to address the question whether variant infectivity and spreading is related to its binding to the receptor.

## Methods

SARS-CoV-2 Spike protein is a class I fusion homotrimer glycoprotein that is composed a total length 1273 residues^56^ and the binding between the virus and the host cell is mediated by the interaction of the protein S receptor binding domain (RBD, located in the S1 domain) with the angiotensin converting enzyme receptor 2 (ACE2). Here, for the sake of simplicity, S protein RBD from SARS-CoV-2 was renamed as RBD_x_, where x represents the identification of each SARS-CoV-2 variant. The initial systems were built considering the coordinates of the RBD complex and the ACE2 (PDB code 6M0J)^33^. The protonation states of the protein residues were defined through the propKa program at pH 7^57^. The amino acids were treated with the ff14SB force field^58^ using TLeap module included in AMBER 16^59^. Each system was solvated using TIP3P water^60^ model in a cubic box with 10.0 Å of the amino acid at the end for all Cartesian directions. Then, each system was neutralized using Na^+^ as contra-ions.

We used four minimization steps with 10,000 cycles for each step, applying minimization first to water, contra-ions and protein, in the last step the minimization was applied to all atoms in the system in order to decrease energy, adjust interactions and decrease contacts with conjugate gradient and steepest descent. The systems were heated linearly from 0 to 300 K (tempi = 0.0; temp0 = 300.0) to avoid excessive and sudden fluctuations of the solute in a time of 5 ns in NVT ensemble employing Langevin dynamics as thermostat (collision frequency of 2 ps) had been used to guarantee a system equilibrium. The SHAKE algorithm^61^ was employed to constraints all bonds involving hydrogen atoms.

First, we have performed 10 ns of Classical molecular dynamics (cMD) simulations to calculate the average dihedral and total potentials energies to be taken as reference for the accelerated molecular dynamics (aMD) simulations. Then 200 ns of aMD simulations was carried out for each system: RBD_WT_–ACE2, RBD_Alpha_–ACE2, RBD_Omicron_–ACE2 and RBD_Delta_–ACE2 complex in NPT essemble.

In general, dynamic properties of proteins cannot be simulated directly using molecular dynamics because of nanosecond time scale limitations^54^, since the systems are trapped in potential energy minima with high free energy barriers for large numbers of computational steps^54^. The aMD is a useful technique for enhancing the sampling during MD simulation^62,63^. This technique is based on the reduction of energy barriers between the different states of a biological system^54,64–66^. The approach employ a modified potential transits from state to state at an accelerated rate, enabling the visit of more structures at energy minima^54,64–66^. In general, 500 ns of aMD simulation can be compared to values calculated from the 1 ms cMD simulation and the experimental values^65,67–70^. For this reason, we have used aMD technique in order to enhance sampling in the protein's conformational space, artificially reducing the energy barriers that separate different states of a given system^54,55,71–74^. Additionally, we used the Bio3D package^75^ to perform the principal component analysis (PCA). The PCs were obtained from the diagonalization of the covariance matrix obtained from the Cartesian coordinates of the superposed Cα atoms of complex structure. To avoid an underestimate of the atomic displacement, an iterated superposition procedure was applied before the PCA, where residues displaying the largest positional differences were excluded at each round until only the invariant ‘core’ residues remained^76–79^.

### Protein–protein binding free energy

In this study, we also evaluated the binding energy differences between the complexes and then the decomposition energy was added to assess the energy contribution of each amino acid during the binding of RBD to ACE2. The binding free energy for the each RBD–ACE2 complex was obtained using:

1 $$\Delta {\text{G}}_{{{\text{bind}}}} = {\text{ G}}_{{{\text{RBD}} - {\text{ACE2}}}} - {\text{ G}}_{{{\text{RBD}}}} {-}{\text{ G}}_{{{\text{ACE2}}}}$$

Here, G_RBD–ACE2_ represent the average over the snapshots of a single trajectory of the MD RBD–ACE2complex, G_RBD_ and G_ACE2_ corresponds to the free energy of RBD and ACE2 protein, respectively. The binding free energy was obtained using MMGBSA method^80,81^ implemented in AMBER 16^59^.

In order to calculate free energy with MMGBSA (Eq. 2) 5000 frames were taken from the 10 ns of MD production using^82–84^:

2 $$\Delta G_{bind,MMGBSA} = \Delta E_{MM} + \Delta G_{sol} - T\Delta S$$

where, $\Delta E_{MM}$ is total gas phase energy (sum of $\Delta E_{internal}$, $\Delta E_{electrostatic}$, and $\Delta E_{vdw}$); $\Delta G_{sol}$ is sum of polar ($\Delta G_{GB}$) and non-polar ($\Delta G_{SA}$) contributions to solvation. It is important to note that the entropy contribution was not included in the calculations due to the difficulty of accurately calculating entropy for a large protein–protein complex^85^. It is also worth to note that the frames were taken from the most stable structure observed in PCA graphics.

## Results and discussion

### Analysis of molecular dynamics of RBD–ACE2 complex

**Figure 1:**
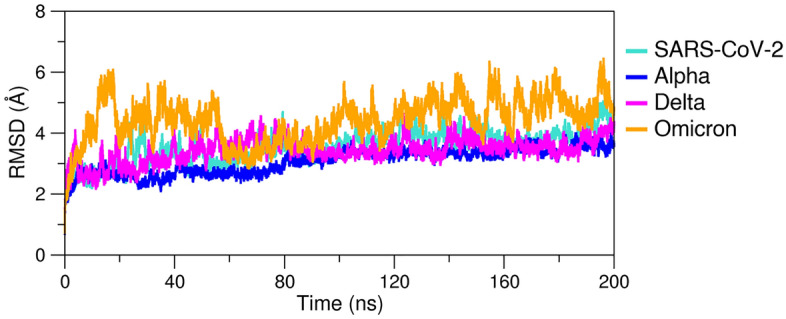
RMSD for RBD_WT_–ACE2, RBD_Alpha_–ACE2, RBD_Omicron_–ACE2 and RBD_Delta_–ACE2 complexes.

All-atom aMD simulations allowed to explore the conformations of protein–protein complex over time for each system: RBD_WT_–ACE2, RBD_Alpha_–ACE2, RBD_Omicron_–ACE2 and RBD_Delta_–ACE2 complex. Figure 1 shows the RMSD during 200 ns of aMD for each system with respect to the reference structure of the equilibrium step. RBD_WT_–ACE2, RBD_Alpha_–ACE2 and RBD_Delta_–ACE2 complexes were within fluctuation in a range of 1–3 Å (Fig. 1), while the RBD_Omicron_–ACE2 complex the present the different variation during simulation in a range of 1–4 Å (Fig. 1). Therefore, the structural equilibrium was reached for all system (Fig. 1).

**Figure 2:**
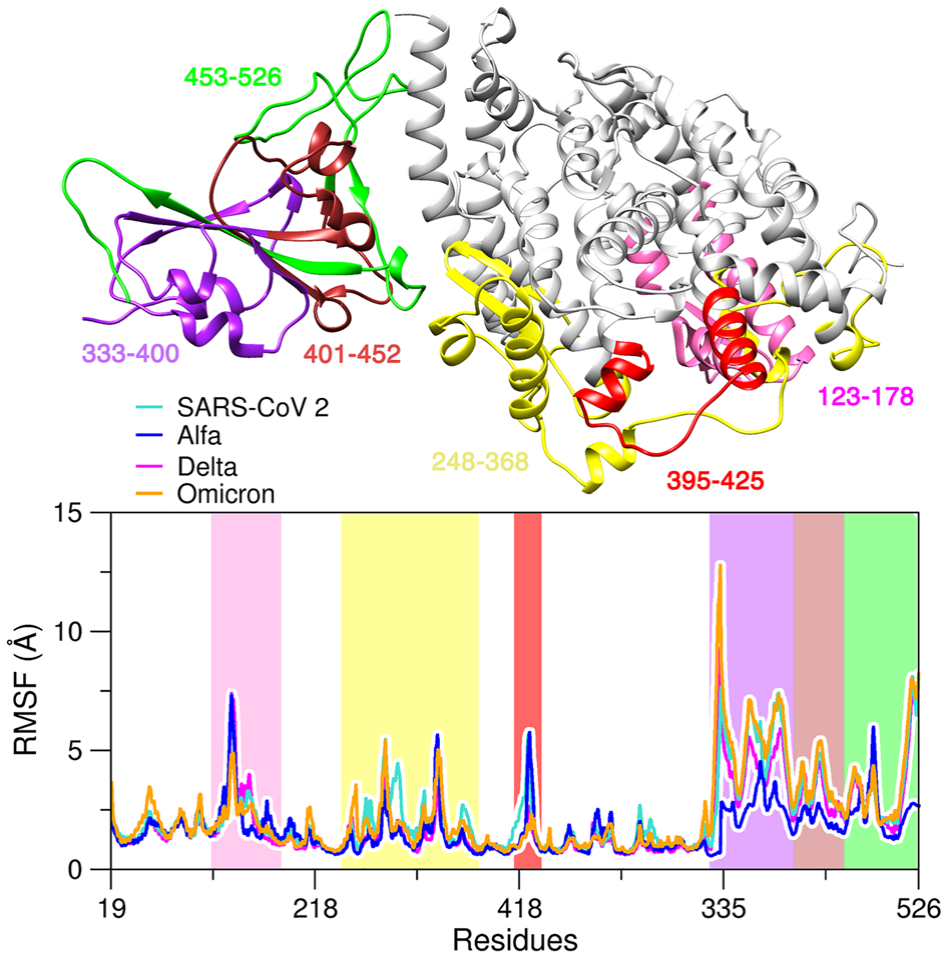
Three-dimensional structure of ACE2 and RBD with RMSF regions for SARS-CoV-2, Alpha, Delta and Omicron systems.

In order to obtain insight into flexibility of each residue in protein–protein complex, we have analyzed the Root-Mean-Square Fluctuations (RMSF) taken into consideration the fluctuations of the backbone atoms. In the RMSF analysis (Fig. 2) ACE2 shows the greatest fluctuation in regions 123 to 178 (in magenta), 395 to 425 (in red) and in the region of residues 248 to 368 (in yellow), that moves to interact with the viral RBD. the RBD_Alpha_ residues show less fluctuation compared to the WT and its last variants (Delta and Omicron).

In this study, we also explore the flexible region in protein–protein complex, through essential dynamics analysis. The PCA graphs, were obtained using the combinations of PC1 vs PC2, PC2 vs PC3 and PC3 vs PC1 (Fig. S2), in which the clusters demonstrate two possible states for all systems in PC1 vs PC2. The color scales represent the trajectory time of the MD, separating the beginning of the structures in the initial time of the final structures of the MD, however, the Alpha variant already has a greater number of clusters, where each time interval is separated into small clusters.

**Figure 3:**
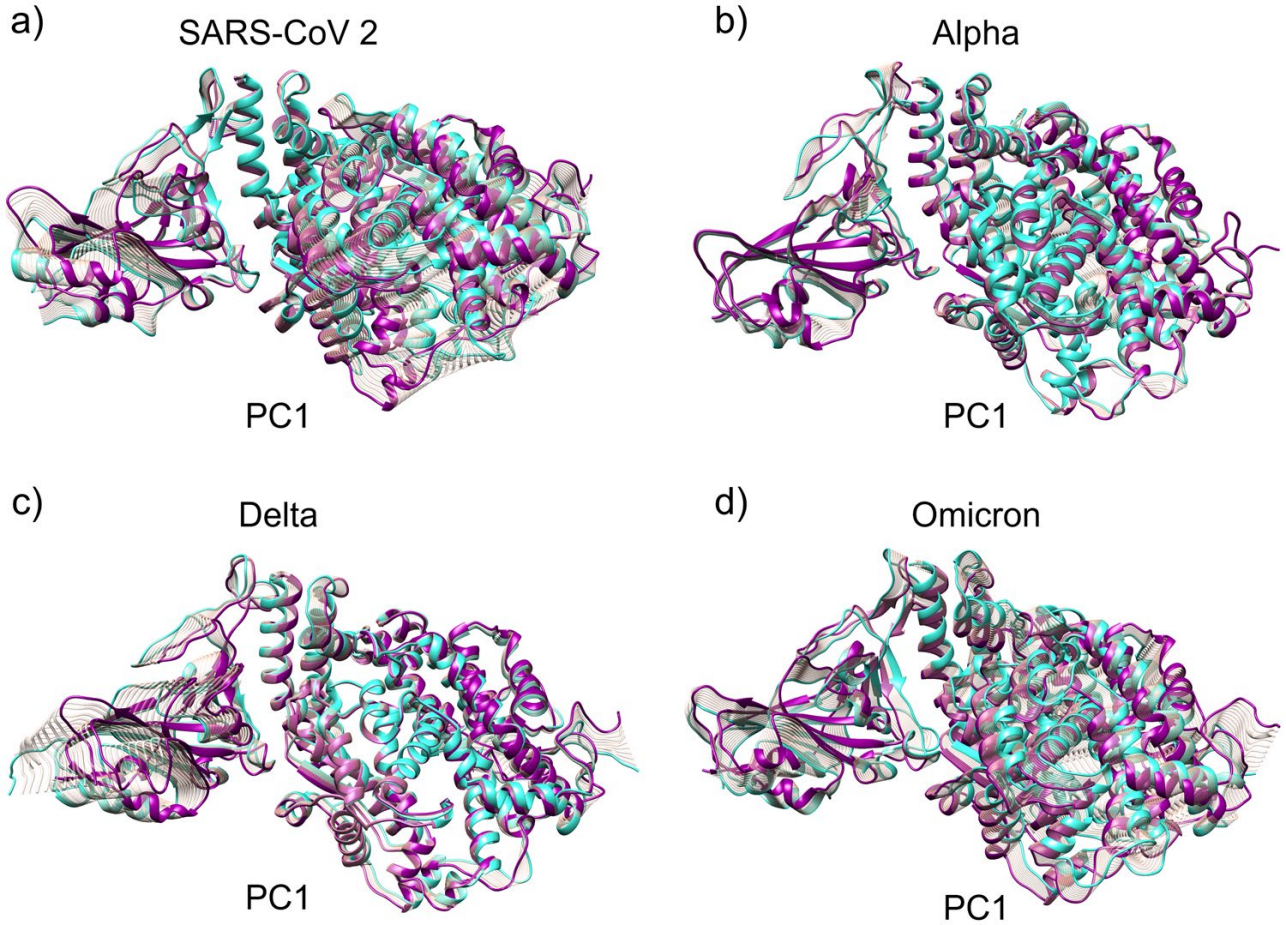
Movements described for the first principal component (PC1) for each structure of ACE2 and RBD. (**a**) Moving in PC1 to the RBD_**WT**_ complex (SARS-CoV-2) and ACE2 receptor. (**b**) changes in PC1 to RBD_Alpha_ and ACE2. (**c**) Change moving of PC1 to RBD_Delta_ and ACE2. (**d**) Moving from PC1 to the complex between RBD_Omicron_ and ACE2. In turquoise, the initial structure of the movement, in dark magenta, the final structure and in gray, the intermediate structures of the movement. The conformational dynamics were obtained from 200 ns of aMD simulations.

For Omicron system the structures are visibly separated into blue structures and red structures (see SI, Fig. S2), indicating that the initial structures differ from the final ones, leading to variations in the aMD structures (Fig. 3). The PCA analysis showed that the RBD_WT_ and the RBD_micron_–RBD_Omicron_ variant present greater conformational fluctuations, however, the RBD_Alpha_ variant stands out for its greater stability. In PC1 there are not many movements in RBD and ACE2 (Fig. 3). The main movement of RBD_WT_ and RBD_micron_–RBD_Omicron_ is similar because they have a greater number of movements. The Spike protein, via RBD, when it binds, causes changes in ACE2, as shown in Fig. 3. The other conformational changes are shown in PC2 and PC3 in Fig. S3 for all systems.

### Binding free energy MMGBSA and decomposition by residue

To assess the affinity of the virus for the human receptor and a possible potential risk of immune evasion by the variants, we calculated the free energy using MM/GBSA [∆G_bind_ (MMGBSA)] based on the points of greatest stability of the aMD trajectory (see Table 1). The RBD_micron_–RBD_Omicron_ shows the highest binding affinity to ACE2, reflecting the infectivity process, but its conformational fluctuations is similar to the other variants. RBD_micron_–RBD_Omicron_ present an adaptive and non-aggressive process when compared to the RBD_Alpha_ (with free energy of binding equal to − 62.7836 kcal/mol), which demonstrated the lower free energy than RBD_WT_ (− 59.7205 kcal/mol). Based on the higher conformational stability of the Alpha variant the high risk is evident and demonstrates a worrying risk of immune evasion due to its degrees of affinity with ACE2.

**Table 1:** Binding free energy for WT systems (SARS-CoV-2) and variants (Alpha/Delta/Omicron).

Energy (kcal/mol)	WT	Alpha	Delta	Omicron
∆E_vdw_	− 95.6 (0.18)	− 107.3 (0.21)	− 103.4 (0.16)	− 96.4 (0.16)
∆Eele	− 625.8 (0.94)	− 608.5 (0.91)	− 955.1 (1.05)	− 1381.7 (1.24)
∆E_GB_	675.0 (0.87)	667.5 (0.87)	1006.3 (1.01)	1416.2 (1.15)
∆E_surf_	− 13.4 (0.02)	− 14.5 (0.02)	− 13.9 (0.02)	− 13.5 (0.02)
∆G_gas_	− 721.3 (0.96)	− 715.8 (0.91)	− 1058.5 (1.09)	− 1478.1 (1.24)
∆G_sol_	661.6 (0.86)	653.0 (0.86)	992.4 (0.99)	1402.7 (1.15)
∆G_bind (MMGBSA)_	− 59.7 (0.28)	− 62.8 (0.23)	− 66.1 (0.21)	− 75.4 (0.23)

The RBD_Delta_ has a higher binding affinity with the human receptor compared to the RBD_WT_ (− 66.1357 kcal/mol), which demonstrates the great concern of infections based on this variant. The high risk of infectivity is pointed out as greater among the variants because they have a more favorable ∆G_bind_ in comparison to RBD_WT_. Therefore, the risk of evolution and emergence of new variants may represent a major health concern due to the degree of affinity that evolves the greater affinity for the human receptor.

**Figure 4:**
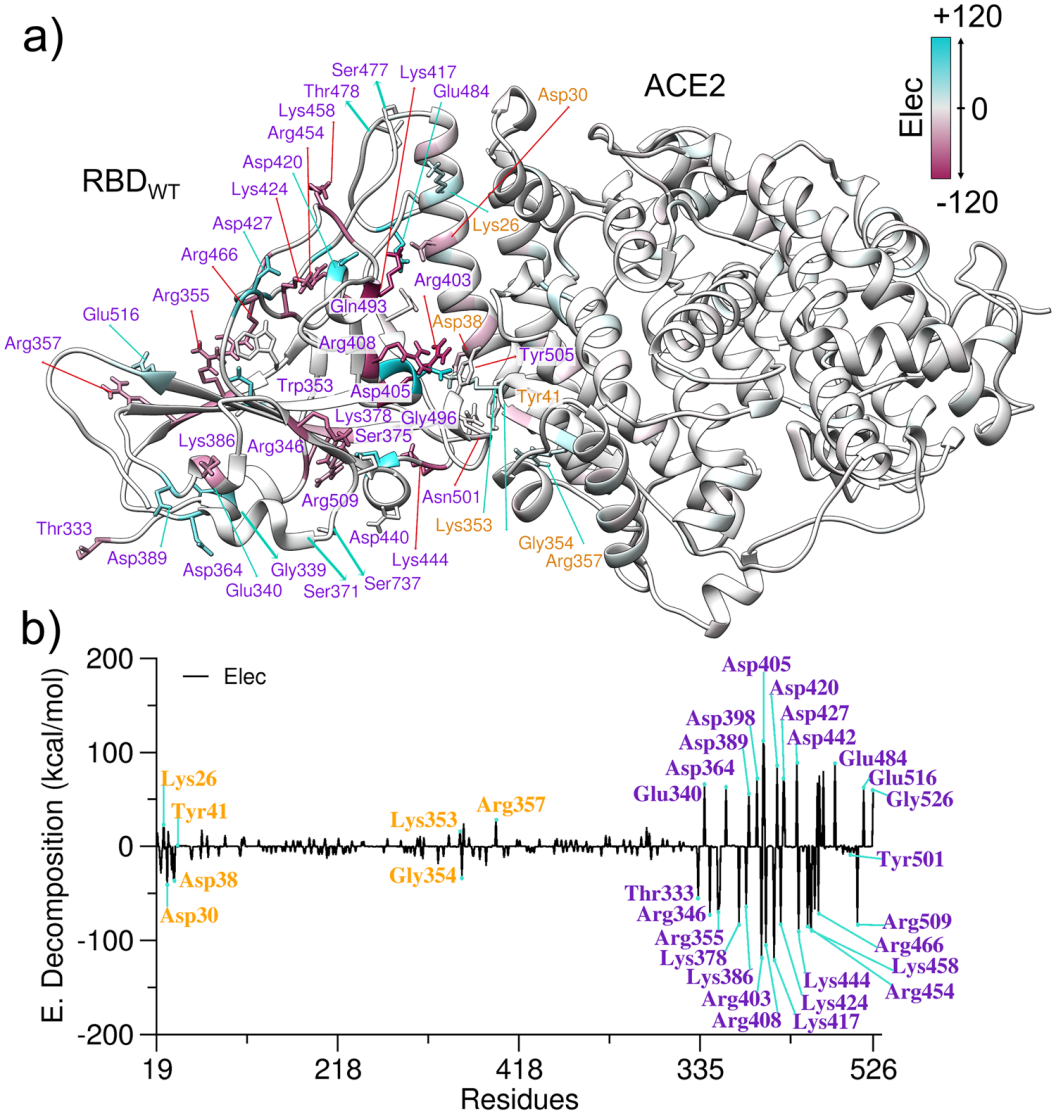
(**a**) Three-dimensional structure of the RBD_WT_ and ACE2 complex with the electrostatic energy regions. (**b**) Decomposition energy per residue for the RBD_WT_ system connected to ACE2. The label in orange is from the ACE2 region and in purple is from RBD.

The effect of mutations can be investigated through the free energy calculations that track the influence of changes in certain positions^49^. The results of the energy of decomposition by residue for RBD_WT_–ACE2, RBD_Alpha_–ACE2, RBD_Omicron_–ACE2 and RBD_Delta_–ACE2 complex demonstrate that the RBD is the region that has more energy variations, attractive and repulsive, when evaluated the electrostatic contributions (see Fig. 4, Figs. S4, S5 and S6). The evaluation of the decomposition energy per residue shows the mutations N440K, T478K, Q493R and Q498R observed in RBD_Omicron_ provide favorable interaction between RBD_Omicron_ and ACE2. Curiously, all these mutations include positively charged residues Lys or Arg (see Table 2). For example, K478 in RBD_Omicron_ present a stabilization effect (− 85.8 kcal/mol), while T478 in RBD_WT_ has a destabilization effect (0.7 kcal/mol), see Table 2. Additionally, Table S2 shows the hydrogen bonds in the protein–protein interaction for the SARS-Cov-2, Alpha, Delta and Omicron system.

**Table 2:** Decomposition energies per residue in kcal/mol for the main mutation positions of RBD WT, Alpha, Delta and Omicron.

SARS-CoV-2		Alpha		Delta		Omicron	
G339	0.7		0.8		0.8	G339D	68.4
S371	0.8		0.4		1.1	S371L	0.8
S373	1.0		0.6		0.9	S373P	0.6
S375	− 0.3		− 0.3		− 0.1	S375F	− 0.4
K417	− 121.2		− 131.5		− 112.4	K417N	− 2.3
N440	− 0.4		− 0.3		− 0.2	N440K	− 98.6
G446	0.3		− 0.2		− 0.2	G446S	1.2
L452	− 0.7		− 0.6	L452R	− 90.5	L452	− 1.4
S477	− 1.1		− 1.7		− 1.4	S477N	− 0.6
T478	0.7		− 2.4	T478K	− 82.6	T478K	− 85.8
E484	88.2		94.4		93.8	E484A	0.1
Q493	− 8.7		− 11.6		− 8.8	Q493R	− 163.7
G496	− 3.6		− 3.1		− 4.9	G496S	− 6.3
Q498	− 6.7		− 2.1		− 7.4	Q498R	− 161.0
N501	− 8.6	N501Y	− 8.1		− 10.1	N501Y	− 2.2
Y505	− 7.4		− 5.5		− 8.0	Y505H	− 1.4

The N501Y mutation in the RBD_Alpha_ has a very similar contribution to the RBD_WT_ system. This mutation does not cause such apparent changes in the energetic contributions. Therefore, its conformational stability is the main feature that contributes to the better binding of RBD_Alpha_ to ACE2, compared to the RBD_WT_. The alterations in the Delta variant cause a highly attractive energy, in which the residue L352R had an energetic contribution of − 90,524 kcal/mol and T478K equal to − 82,654 kcal/mol (see Table 2), indicating that there is a great improvement in the binding with the receptor. The mutations present in RBD_Omicron_ demonstrate that during the gain in the energetic contribution of the residues.

Some mutations present in RBD_Omicron_ (N440K, T478K, Q493R, Q498R) demonstrate that substitutions for positively charged residues guide an improvement in the contribution to the interaction with ACE2 (Fig. S7). T478K is located in a more solvent-oriented region, allowing interaction with ACE2, due to the increase in the side chain Fig. S7a. As well, the Q493R substitution allows favorable interaction with negatively charged residues of ACE2 such as Asp38 and Glu35, improving the binding with the receptor and increasing the affinity of the Spike protein (Fig. S7b). The N440K in the micron Omicron is located in the region most focused on the solvent, increasing the contribution of this region with the medium (Fig. S7c), whereas the Q498R substitution improves the protein–protein interaction since this contribution is 24 times greater in relation to the WT, demonstrating that these substitutions are essential for improving interaction with ACE2 (Fig. S7d).

A recent study has suggested that RBD_Omicron_ present a slightly reduced binding to ACE2 compared to RBD_WT_ (RBD of the original Wuhan strain)^86^ and RBD_Delta_. The EC_50_ values were determined to be 120, 150 and 89 ng/mL for RBD_WT_, RBD_Omicron_ and RBD_Delta_, respectively^86^. Other experimental study have proposed that RBD_Omicron_ shows weaker binding affinity than RBD_Delta_ to ACE2^87^. Han and coworkers have measured the binding affinities of the RBDs to ACE2 with surface plasmon resonance (SPR) assay^88^. They found that RBD_WT_, RBD_Omicron_ and RBD_Delta_ binds to ACE2 with a dissociation constant (K_D_) of 24.63 nM, 31.40 and 25.07. Other experimental study shows that RBD_Omicron_ and RBD_Delta_ binds to ACE2 at a similar affinity to that of the RBD_WT_^89^.

On the other hand, Lin and coworkers have obtained kinetic-affinity values of 87.9 nM for RBD_WT_ and 40.8 nM for RBD_Omicron_. These values highlight ~ 2.2-fold-enhanced receptor-binding with RBD_Omicron_^90^. A recent computational study have investigated the interaction between the RBD of both the WT and Omicron variant of SARS-CoV-2 with the ACE2 receptor using molecular dynamics and molecular mechanics-generalized Born surface area (MM-GBSA)-based binding free energy calculations^51^. Authors have carried out 100 ns of MD simulations for each complex and have suggested that the RBD_Omicron_ has an increased affinity for the ACE2 receptor in comparison to RBD_WT_^51^. This last study has a closer relationship to our strategy used in here. The main difference is that we are describing computational result from 200 ns of aMD to explore molecular details interactions that occur in the Spike RBD of Alpha, Delta and Omicron variants in the binding with the ACE2 receptor. It is important to note that some bioinformatic models predicted an increase in the ACE2 binding affinity of RBD_Omicron_^91^. Here, our results are suggesting that complexes studied have similar fluctuations and that mutations present in RBD_Omicron_, RBD_Delta_ and RBD_Alpha_ increase the binding to ACE2 compared to RBD_WT_.

## Conclusion

In this study, we evaluated the effect of residues mutation on structural and energetics of Spike protein RBD from SARS-CoV-2 variants in complex with the human ACE2 receptor. All-atoms accelerated Molecular Dynamics simulations and PCA analysis shows that that the RBD_Omicron_–ACE2 complex present similar fluctuation in comparison to S protein from WT, Delta and Alpha variants. The binding affinity of each RBD_x_ to ACE2 was obtained using MM-GBSA methods. The results show that the trend in the calculated binding free energies correlates well with virus infectivity of each variant. The mutation in RBD_Omicron_ increase the affinity of Spike protein for ACE2 and may explain Omicron's high transmissibility in comparison with other SARS-CoV-2 variants. The stabilization effect RBD_Omicron_–ACE2 complex is achieved manly due the substitution of uncharged residues by positively charged residues: Lys and Arg in key positions. Overall, our results may explain at molecular level the effect of key mutations in the Spike protein for virus infectivity.

## Supplementary Information

Supplementary Information.

## Data Availability

All necessary files to conduct this work (.pdb and .parm7) can be found attached as the Supporting Information. The AMBER18 suite of programs and the Amber ff14SB force field were used to carry out the MD simulations and can found at https://ambermd.org/.
