# Supplementary material for: Assessment of mutations on RBD in the Spike protein of SARS-CoV-2 Alpha, Delta and Omicron variants
Source: Sci Rep. 2022 May 20;12:8540. doi: 10.1038/s41598-022-12479-9 (PMC9121086; doi:10.1038/s41598-022-12479-9)
Supplement: Supplementary file 1 — Supplementary Information. [file 41598_2022_12479_MOESM1_ESM.docx]

**Assessment of Mutations on RBD in the Spike Protein of SARS-CoV‑2 Alpha, Delta and Omicron Variants**

Clauber Henrique Souza da Costa^1^, Camila Auad Beltrão de Freitas^1^, Cláudio Nahum Alves^1^, Jerônimo Lameira^1*^

*^1^Laboratório de Planejamento e Desenvolvimento de Fármacos.* *Universidade Federal do Pará. Rua Augusto Correa S/N. Belém-PA.*

**ORCID of the authors:**

Clauber Henrique Souza da Costa: 0000-0002-6915-1056.

Camila Auad Beltrão de Freitas: 0000-0003-1765-7925.

Cláudio Nahum Alves: 0000-0001-6576-4229.

Jerônimo Lameira: 0000-0001-7270-1517.

**Supporting Information**

The conformational dynamics of the protein can be understood using a wide variety of dynamic processes resulting from a trajectory, and one of the first steps for aMD is the calculation of the variables ​​total potential energy (EPTOT) and dihedral angle energy (DIHED), as can be seen in table S1, which presents the parameters used for the simulation of aMD, computed during 10ns of cMD, where the variables ​​EPTOT and DIHED demonstrated stability. The EPTOT for the SARS-CoV-2 system and the Alpha and Omicron variants have similar dihedral variations throughout the classical simulation, while the EPTOT energy of the SARS-Cov-2 has a greater variation compared to other systems (Figure S1a). The mean values ​​of EPTOT and DIHED can be found in Table S1 and these parameters are used as input to aMD. Figure S1 shows the information collected during aMD to define the parameters needed for aMD. Thus, we obtain for the SARS-CoV-2, Alpha and Omicron systems the average total potential energy of - 307852,225; -311397.0093 and -311012.6842 kcal/mol, respectively. We also obtained an average dihedral energy of 10452.9602; 10457.7996 and 10478.9487 kcal / mol, for the SARS-CoV-2, Alpha and Omicron systems, respectively.

Using this information, also considering that the spike protein RBD and ACE2 complex has 591 residues (according PDB 6M0J model) and system has variation in the number of atoms due to the substitutions in the variants, we used the simulation parameters of 200ns in aMD for each studied system.

Table S1: Variables and parameters used in the aMD simulation for all systems, obtained during cMD

| System | **Variables** | | | | **Calculated Parameters** | | | |
| --- | --- | --- | --- | --- | --- | --- | --- | --- |
|  | EPtot  (kcal/mol) | DIHED  (kcal/mol) | Total  Atoms | Residues | Ethreshd | Alphad | Ethreshp | Alphap |
| SARS -CoV-2 | -307852.2225 | 10452.9602 | 102041 | 791 | 13616.9602 | 632.8 | -291525.6625 | 16326,56 |
| Alfa | -311397.0093 | 10457.7996 | 103191 | 791 | 13621.7996 | 632.8 | -294886.4493 | 16510.56 |
| Delta | -309646.8763 | 10454.8918 | 102616 | 791 | 13618.8918 | 632.8 | -293228.3163 | 16418.56 |
| Omicron | -311012.6842 | 10478.9487 | 103159 | 791 | 13642.9487 | 632.8 | -294507.2442 | 16505.44 |


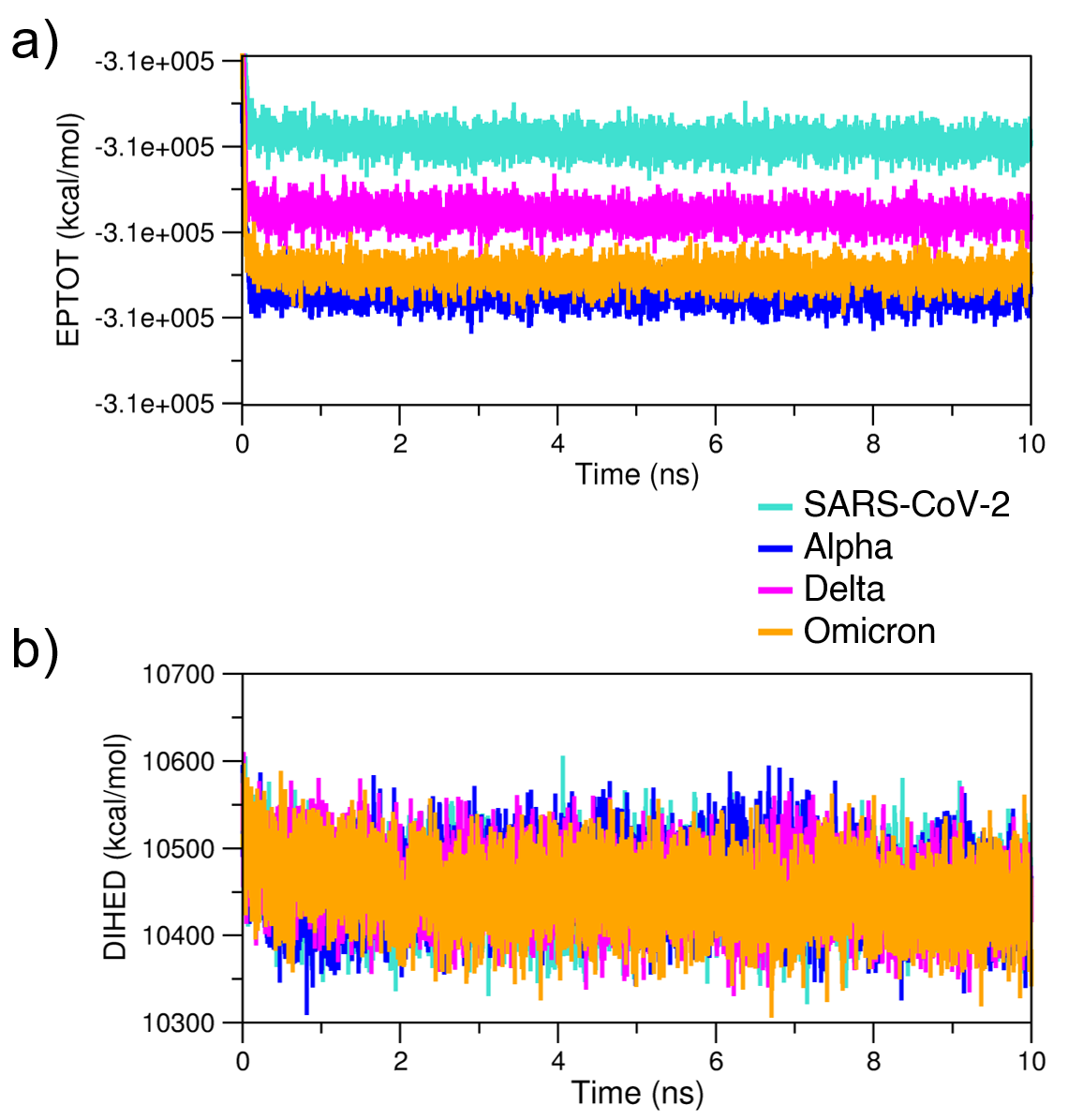


Figure S1: **A)** Total potential energy (EPTOT) and **B)** DIHED during 10ns of cMD for the complex between RBD of protein spike variants and ACE2.


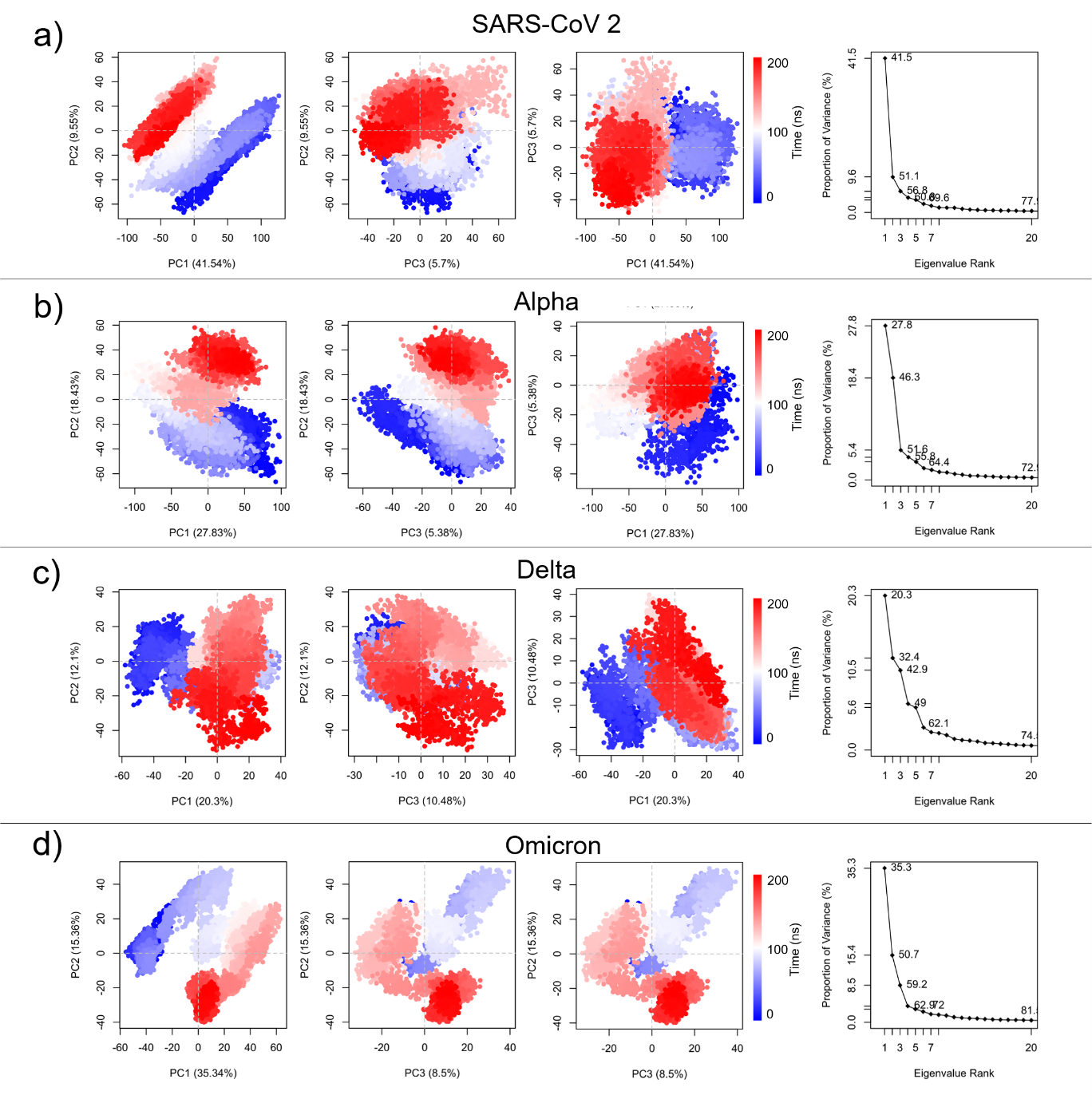


Figure S2: PCA (PC1 vs PC2, PC2 vs PC3, and PC1 vs PC3. The colors are according to the simulation time of the MD: the red color indicates the initial nanoseconds of simulation and the blue color indicates the final nanoseconds.


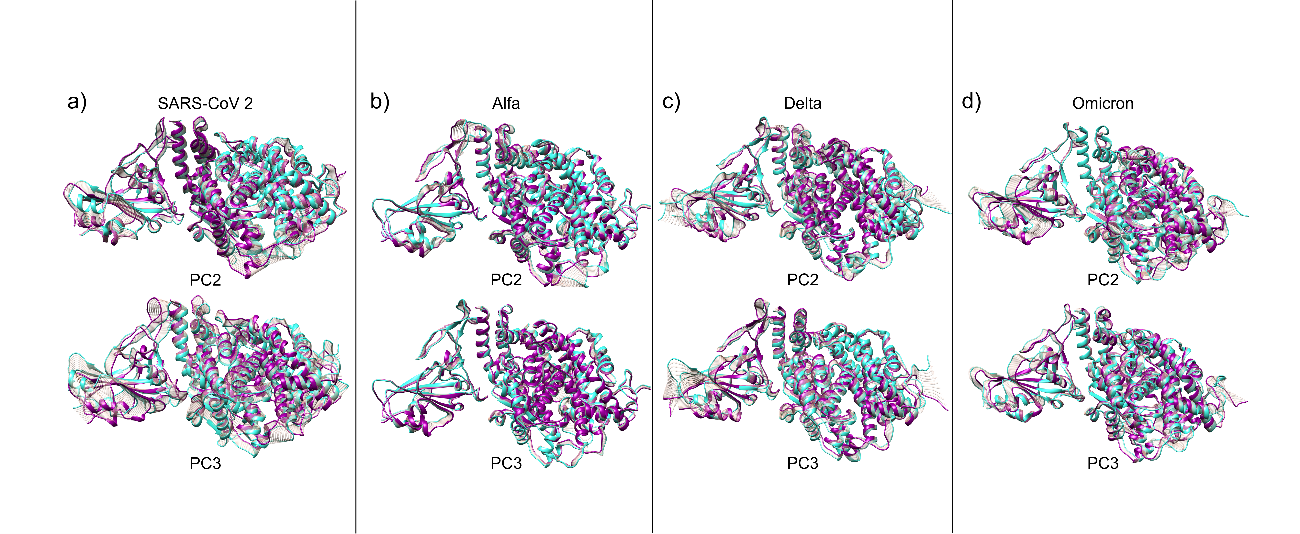


Figure S3: Movements described for the second and third principal components, PC2 and PC3, respectively, for each structure of ACE2 and RBD. A) Native RBD (SARS-CoV-2) and ACE2 receptor. B) RBD_Alpha_ and ACE2 receptor. C) RBD_Delta_ and ACE2 to PC1 and PC2. D) complex between RBD_Omicron_ and ACE2. In turquoise, initial structure of the movement, in dark magenta, final structure and in gray, intermediate structures of the movement. The conformational dynamics were obtained from 200 ns of aMD simulations.


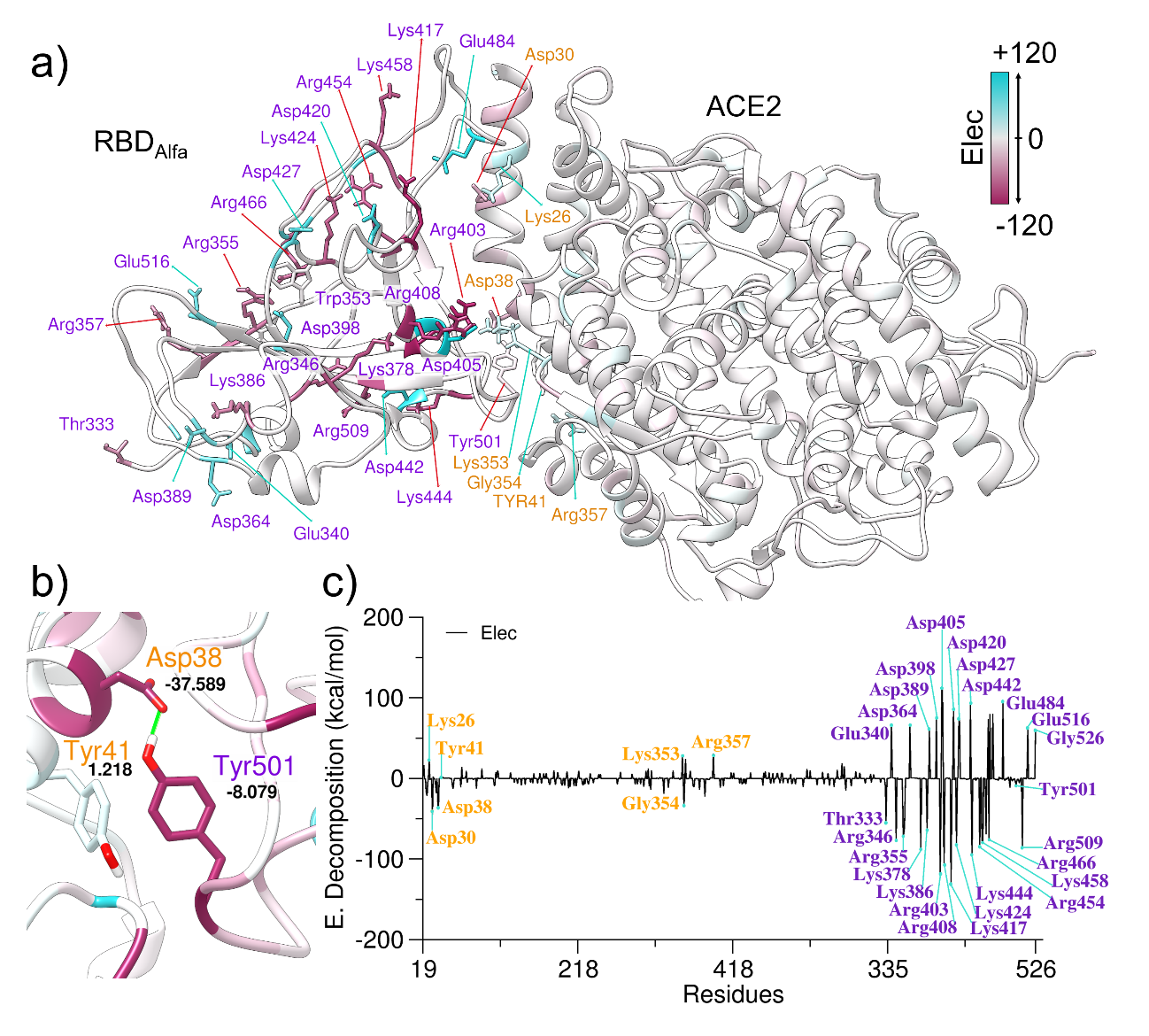


Figure S4: a) three-dimensional structure of the RBD_Alpha_ and ACE2 complex with the electrostatic energy regions. B) Main hydrogen bond of Tyr501 that characterizes the Alpha variant with electrostatic energies. C) Decomposition energy per residue for the RBD_Alpha_ system connected to ACE2. The label in orange is from the ACE2 region and in purple is from RBD.


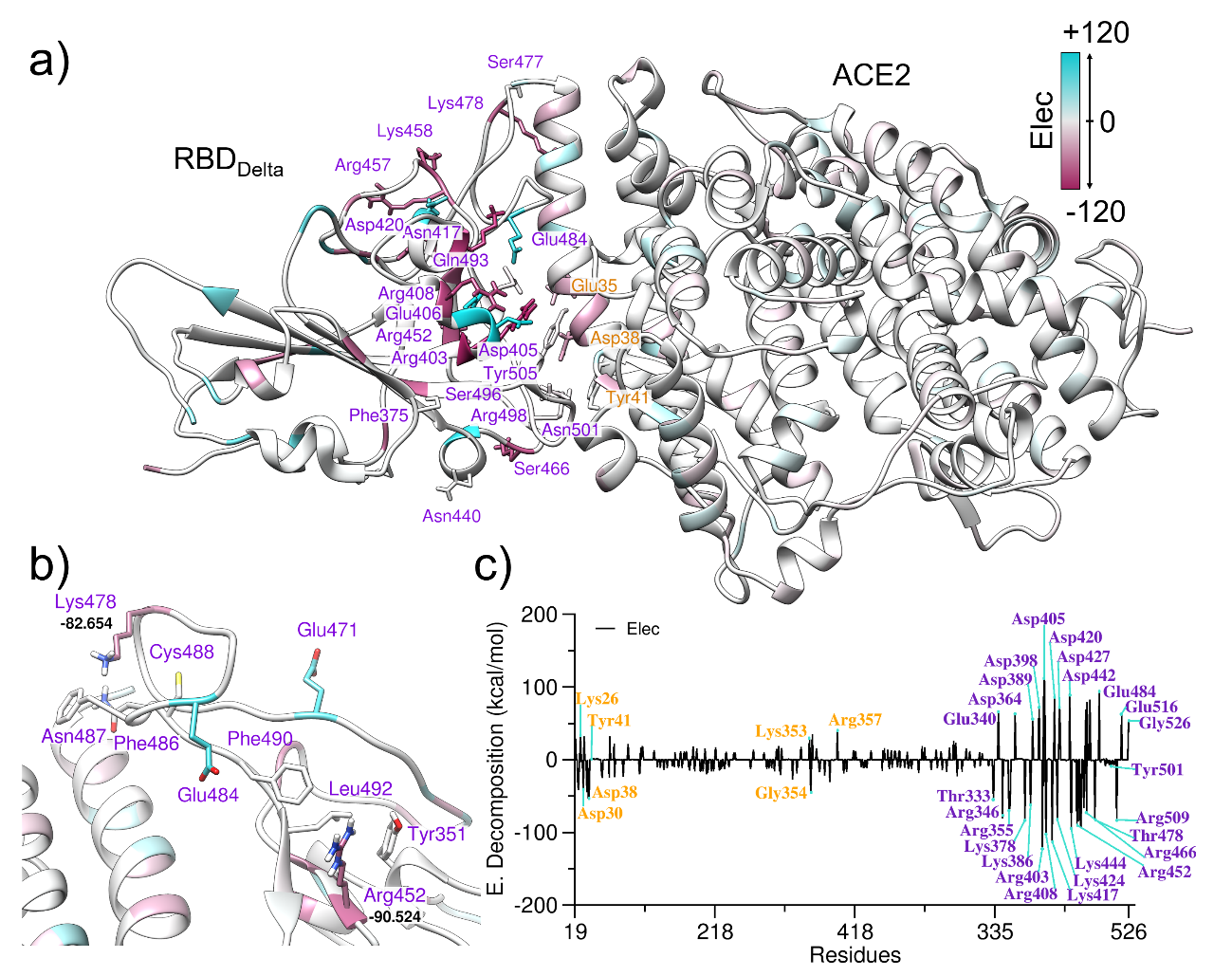


Figure S5: a) three-dimensional structure of the RBD_Delta_ and ACE2 complex with the electrostatic energy regions. B) Change and location of the L452R and T478K that characterize the Delta variation. C) Decomposition energy per residue for the RBD_Delta_ system connected to ACE2. The label in orange is from the ACE2 region and in purple is from RBD.


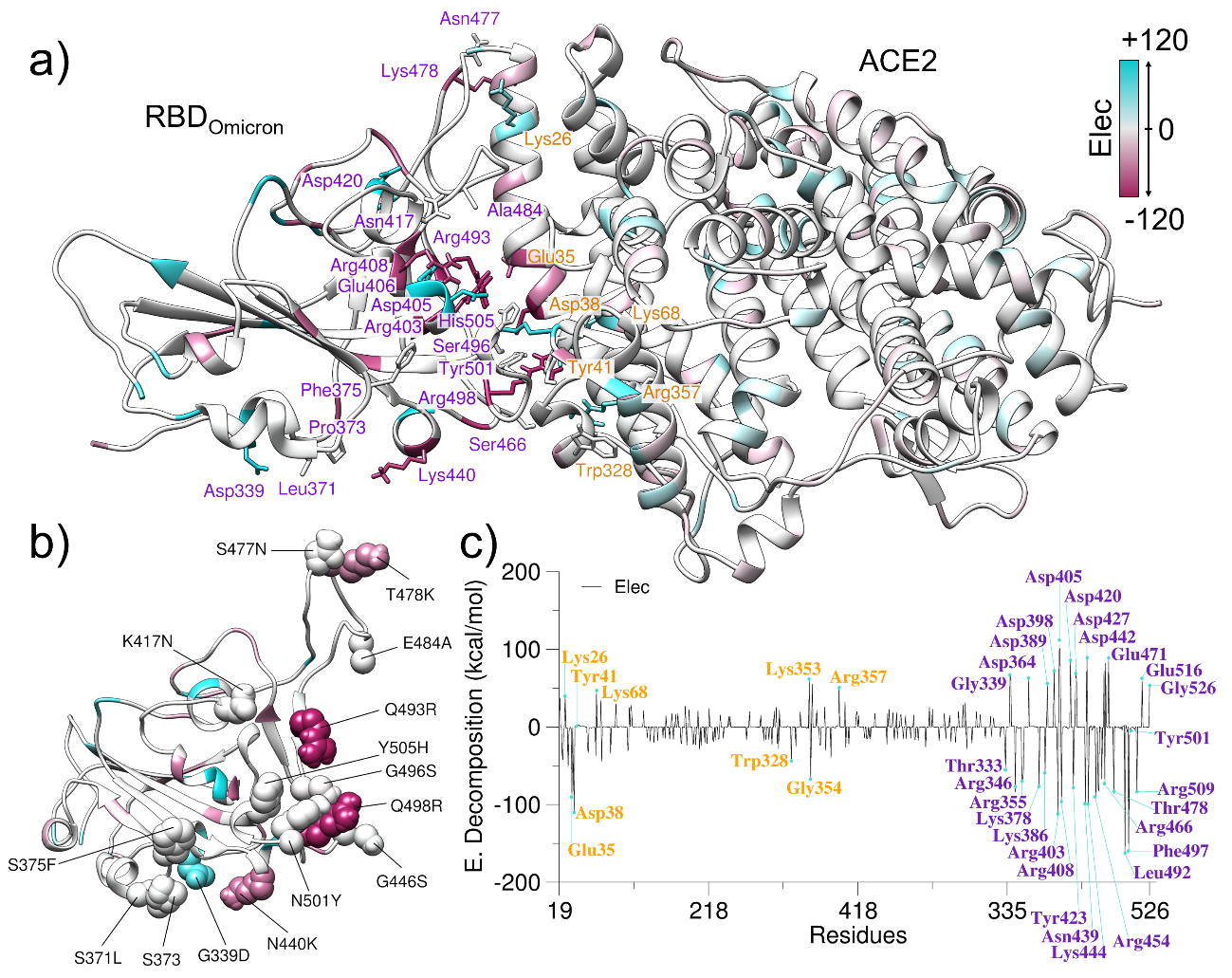


Figure S6: a) three-dimensional structure of the RBD_Omicron_ and ACE2 complex with the electrostatic energy regions. B) Change and location of the residues mutant to Omicron variation. C) Decomposition energy per residue for the RBD_Omicron_ system connected to ACE2. The label in orange is from the ACE2 region and in purple is from RBD.


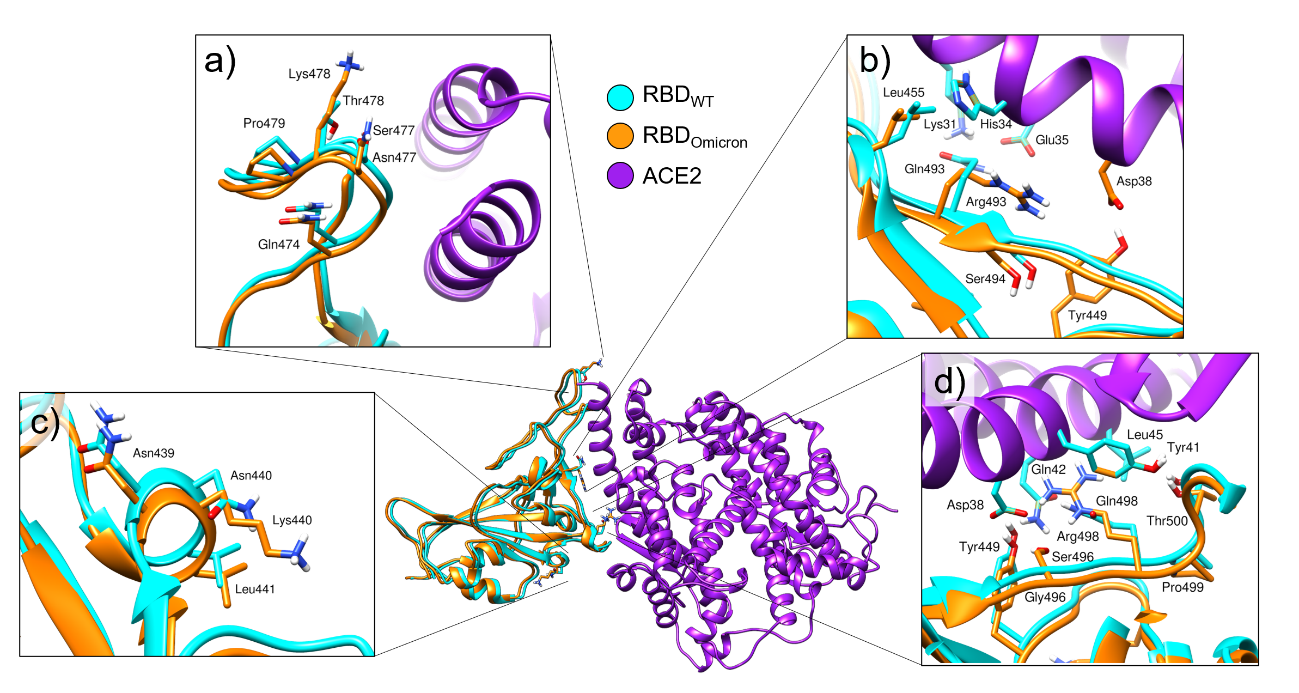


Figure S7: Location of mutations N440K, T478K, Q493R, Q498R in RBD_Omicron_ (in orange) in relation to the RBD structure of SARS-Cov-2 (in cyan)

Table S2: Protein-Protein interaction H_bond between Spike protein RBD and ACE2 receptor, from SARS-CoV-2 and Variant

| Average Distances (Å) | | | | | | | | | |
| --- | --- | --- | --- | --- | --- | --- | --- | --- | --- |
| ACE | RBD | SARS-CoV-2 | | Alfa | | Delta | | Omicron | |
|  |  | Occ^a^ | Dist^b^ | Occ^a^ | Dist^b^ | Occ^a^ | Dist^b^ | Occ^a^ | Dist^b^ |
| Gln24-HE22 | Phe486-O | 40.2 | 2.84 |  |  | 15.8 | 2.84 |  |  |
| Gln24-O | Tyr489-HH |  |  |  |  |  |  | 16.7 | 2.81 |
| Gln24-OE1 | Asn487-H |  |  |  |  | 13.5 | 2.86 |  |  |
| Gln24-OE1 | Asn490-H |  |  |  |  |  |  | 15.5 | 2.86 |
| Lyn31-NZ | Phe490-H |  |  |  |  |  |  | 15.6 | 2.91 |
| Lys31-HZ2 | Gln493-OE1 | 10.2 | 2.79 |  |  | 15.2 | 2.79 |  |  |
| Lys31-HZ3 | Gln493-OE1 | 10.9 | 2.79 |  |  |  |  |  |  |
| Lys31-HZ1 | Gln493-OE1 | 10.7 | 2.79 |  |  | 15.0 | 2.79 |  |  |
| His34-NE2 | Tyr453-HH |  |  | 14.9 | 2.84 |  |  |  |  |
| Glu35-OE1 | Gln493-HE21 |  |  | 12.2 | 2.83 | 14.6 | 2.83 |  |  |
| Glu35-OE1 | Gln493-HE22 | 14.2 | 2.83 |  |  | 12.5 | 2.83 |  |  |
| Glu35-OE2 | Gln493-HE21 |  |  | 11.3 | 2.83 | 13.0 | 2.83 |  |  |
| Glu35-OE2 | Gln493-HE22 | 14.1 | 2.83 |  |  | 12.3 | 2.83 |  |  |
| Glu35-OE2 | Arg493-HH11 |  |  |  |  |  |  | 14.1 | 2.80 |
| Glu35-OE1 | Arg493-HH22 |  |  |  |  |  |  | 14.1 | 2.80 |
| Glu35-OE1 | Arg493-HH11 |  |  |  |  |  |  | 14.1 | 2.80 |
| Glu35-OE2 | Arg493-HH22 |  |  |  |  |  |  | 14.0 | 2.80 |
| Glu35-OE2 | Arg493-HH21 |  |  |  |  |  |  | 11.3 | 2.80 |
| Glu35-OE1 | Arg493-HH21 |  |  |  |  |  |  | 10.8 | 2.80 |
| Glu35-OE2 | Arg493-HH12 |  |  |  |  |  |  | 17.2 | 2.80 |
| Glu35-OE1 | Arg493-HH12 |  |  |  |  |  |  | 18.5 | 2.80 |
| Glu37-OE1 | Tyr505-HH | 25.6 | 2.72 |  |  |  |  |  |  |
| Glu37-OE2 | Tyr505-HH | 38.9 | 2.71 |  |  | 17.8 | 2.70 |  |  |
| Asp38-OD1 | Ser496-HG |  |  |  |  |  |  | 38.7 | 2.69 |
| Asp38-OD2 | Ser496-HG |  |  |  |  |  |  | 35.9 | 2.69 |
| Asp38-OD1 | Gln498-HE21 | 10.7 | 2.83 |  |  |  |  |  |  |
| Asp38-OD1 | Arg498-HH11 |  |  |  |  |  |  | 15.1 | 2.80 |
| Asp38-OD1 | Arg498-HH12 |  |  |  |  |  |  | 20.8 | 2.79 |
| Asp38-OD1 | Arg498-HH21 |  |  |  |  |  |  | 19.3 | 2.79 |
| Asp38-OD1 | Arg498-HH22 |  |  |  |  |  |  | 18.4 | 2.80 |
| Asp38-OD2 | Arg498-HH12 |  |  |  |  |  |  | 19.0 | 2.79 |
| Asp38-OD2 | Arg498-HH22 |  |  |  |  |  |  | 18.2 | 2.80 |
| Asp38-OD2 | Arg498-HH11 |  |  |  |  |  |  | 17.1 | 2.80 |
| Asp38-OD2 | Arg498-HH21 |  |  |  |  |  |  | 17.0 | 2.80 |
| Asp38-OD2 | Gln498-HE22 | 11.3 | 2.83 |  |  |  |  |  |  |
| Asp38-OD1 | Tyr449-HH | 36.8 | 2.71 | 19.0 | 2.69 | 19.5 | 2.70 | 19.1 | 2.71 |
| Asp38-OD2 | Tyr449-HH | 37.0 | 2.70 | 16.5 | 2.70 | 23.0 | 2.70 | 20.8 | 2.71 |
| Asp38-OD2 | Tyr501-HH | 0.0 |  | 25.6 | 2.67 |  |  |  |  |
| Asp38-OD1 | Tyr501-HH | 0.0 |  | 32.0 | 2.66 |  |  |  |  |
| Ala386-O | Tyr505-HH | 0.0 |  | 29.1 | 2.73 |  |  |  |  |
| Tyr41-OH | Thr500-HG1 | 19.4 | 2.80 |  |  |  |  |  |  |
| Tyr83-HH | Gly453-O | 0.0 |  | 15.4 | 2.76 |  |  |  |  |
| Tyr83-HH | Gly485-O | 0.0 |  |  |  |  |  | 38.3 | 2.76 |
| Tyr83-HH | Phe486-O | 18.6 | 2.80 |  |  | 24.8 | 2.76 |  |  |
| Tyr83-OH | Tyr489-HH | 0.0 |  |  |  |  |  | 10.2 | 2.83 |
| Lys353-O | Gly502-H | 67.6 | 2.86 | 60.3 | 2.87 | 52.0 | 2.87 | 47.5 | 2.87 |
| Lys353-HZ1 | Gln498-OE1 | 19.0 | 2.80 |  |  |  |  |  |  |
| Lys353-HZ1 | Gly496-O | 12.7 | 2.80 |  |  |  |  |  |  |
| Lys353-HZ2 | Gln498-OE1 | 18.3 | 2.80 |  |  |  |  |  |  |
| Lys353-HZ2 | Gly496-O | 12.5 | 2.80 |  |  |  |  |  |  |
| Lys353-HZ3 | Gly496-O | 12.3 | 2.80 |  |  |  |  |  |  |
| Lys353-HZ3 | Gln498-OE1 | 19.4 | 2.80 |  |  |  |  |  |  |
| Asp355-OD1 | Thr500-HG1 |  |  | 42.5 | 2.70 | 61.8 | 2.69 | 87.1 | 2.70 |
| Asp335-OD2 | Thr500-HG1 |  |  | 38.3 | 2.69 |  |  |  |  |
| Asp355-OD1 | Thr505-HG1 | 22.8 | 2.70 |  |  |  |  |  |  |
| Asp355-OD2 | Thr505-HG1 | 33.3 | 2.69 |  |  | 18.1 | 2.71 |  |  |
| Ala386-O | Thr505-HH |  |  |  |  | 23.2 | 2.72 |  |  |

^a^ % Occupancy, interaction occupancy rate over time of 200ns aMD

^b^ distance in Å
